# Electrochemical Sensor for Hydrogen Leakage Detection at Room Temperature

**DOI:** 10.3390/s25010264

**Published:** 2025-01-05

**Authors:** Gimi Aurelian Rîmbu, Lucian Pîslaru-Dănescu, George-Claudiu Zărnescu, Carmen Alina Ștefănescu, Mihai Iordoc, Aristofan Alexandru Teișanu, Gabriela Telipan

**Affiliations:** 1 Department of Energy, Environment and Climate Change, National Institute for Research and Development in Electrical Engineering ICPE-CA, 030138 Bucharest, Romania; gimi.rimbu@icpe-ca.ro (G.A.R.); carmen.stefanescu@icpe-ca.ro (C.A.Ș.); mihai.iordoc@icpe-ca.ro (M.I.); aristofan.teisanu@icpe-ca.ro (A.A.T.); 2 Laboratory of Sensors/Actuators and Energy Harvesting, National Institute for Research and Development in Electrical Engineering ICPE-CA, 030138 Bucharest, Romania; george.zarnescu@icpe-ca.ro (G.-C.Z.); gtelipan@yahoo.com (G.T.)

**Keywords:** sensing material, sensing element, signal conditioning, response time, hydrogen sensor

## Abstract

The use of hydrogen as fuel presents many safety challenges due to its flammability and explosive nature, combined with its lack of color, taste, and odor. The purpose of this paper is to present an electrochemical sensor that can achieve rapid and accurate detection of hydrogen leakage. This paper presents both the component elements of the sensor, like sensing material, sensing element, and signal conditioning, as well as the electronic protection and signaling module of the critical concentrations of H_2_. The sensing material consists of a catalyst type Vulcan XC72 40% Pt, from FuelCellStore, (Bryan, TX, USA). The sensing element is based on a membrane electrode assembly (MEA) system that includes a cathode electrode, an ion-conducting membrane type Nafion 117, from FuelCellStore, (Bryan, TX, USA). and an anode electrode mounted in a coin cell type CR2016, from Xiamen Tob New Energy Technology Co., Ltd, (Xiamen City, Fujian Province, China). The electronic block for electrical signal conditioning, which is delivered by the sensing element, uses an INA111, from Burr-Brown by Texas Instruments Corporation, (Dallas, TX, USA). instrumentation operational amplifier. The main characteristics of the electrochemical sensor for hydrogen leakage detection are operation at room temperature so it does not require a heater, maximum amperometric response time of 1 s, fast recovery time of maximum 1 s, and extended range of hydrogen concentrations detection in a range of up to 20%.

## 1. Introduction

Hydrogen plays a crucial role in various industries, serving as a feedstock in refineries, a chemical reactant in industrial processes, and a fuel for space rockets [1]. Given the global aim of achieving “Carbon Neutral” status, hydrogen energy has become increasingly vital in the energy transition and is making a significant impact worldwide. In the realm of new energy transportation, hydrogen fuel is assuming an ever-more important role in daily life [2]. The key to establishing a hydrogen energy-based society lies in efficiently producing hydrogen without carbon emissions. However, the use of hydrogen presents safety challenges due to its flammability and explosive nature, combined with its lack of color, taste, and odor [3].

To mitigate potential hazards, especially the risk of explosions, strict monitoring of hydrogen leakage when using hydrogen fuel is essential [4]. Consequently, rapid and accurate detection of hydrogen leakage is a critical step to ensure its safe usage. Gas sensors have proven effective for this purpose, like sensors with semiconductors, catalytic combustion, thermoelectric, and electrochemical sensors [5,6,7,8,9,10,11,12,13]. Among these gas sensors, the electrochemical (potentiometric and amperometric) hydrogen sensors employing solid electrolytes show great promise due to their compact design, straightforward detection mechanism, and high selectivity [3].

Electrochemical gas sensors have gained popularity due to their advantages, including low power consumption [14], high sensitivity, and excellent selectivity [15]. In the case of hydrogen electrochemical sensors, the catalyst characteristics play a crucial role. These catalysts not only directly influence the sensor’s sensitivity but also significantly contribute to the overall sensor cost.

Platinum stands out as the most efficient electrocatalyst, facilitating hydrogen-related electrochemical reactions by reducing the activation energy for hydrogen dissociation [16,17]. Its high catalytic activity for hydrogen has led to its widespread use as a metal electrode in sensor design. For instance, A. V. Kroll introduced an electrochemical gas sensor utilizing platinum as the sensitive electrode back in 1994, demonstrating favorable performance in detecting hydrogen and hydrogen sulfide [18]. However, platinum is a precious metal, and extensive use of it as a sensor electrode can result in higher manufacturing costs for the sensor devices. Consequently, researchers are actively exploring the substitution of platinum metal electrodes with platinum catalyst-modified electrodes to address this cost-related challenge and enhance sensor development [19].

Moreover, the use of platinum in gas sensors also comes with certain drawbacks, such as the self-aggregation of metal particles, which can reduce the active surface area available for interactions between gases and catalyst powders, making the sensor less effective. Additionally, noble metals are expensive, contributing to the high cost of the sensors, and they may exhibit poor selectivity in certain applications. To overcome these shortcomings, researchers have explored various approaches, with one promising solution being the deposition of metal nanoparticles (NPs) onto supportive materials. Among these materials, alternative carbon-based materials like carbon blacks, ordered mesoporous carbons, carbon nanotubes, and carbon nanofibers have shown success in supporting metal NPs for use in electrochemical catalysis and fuel cells. These carbon materials offer excellent conductivity and porous microstructure, which allow for efficient dispersion of the metal particles [20,21,22,23,24,25,26,27,28]. Particular attention has been focused on carbon supports for homogeneous deposition of platinum nanoparticles due to their unique surface structures, exceptional mechanical properties, high electrical conductivity, and large surface areas [29,30]. For example, Y.C. Liu et al. [31] developed a solid-state H_2_ sensor using platinum particles supported on activated carbon powders through the indirect wetting method, achieving a sensitivity of 0.716 A/ppm at a Pt loading of 3.0 mg/cm^2^. Likewise, B. Zhao et al. [28] utilized the incipient wetness impregnation and electrodeposition methods to create a novel electrocatalyst, consisting of Pt NPs supported on activated carbon fibers, for the electrocatalytic hydrogenation of furfural to furfuryl alcohol.

In this study, platinum (Pt) nanoparticles supported on a carbon substrate are used as composite materials to design an electrolyte polymer-based amperometric hydrogen sensor. This paper is divided into two main chapters, each addressing distinct aspects of this study. In the first chapter, the manufacturing process, materials, and membrane–electrode assembly (MEA) are described. This chapter introduces a novel design of an electrochemical sensor for hydrogen leakage detection optimized for operation at room temperature without requiring external heating. The sensor employs advanced materials, including a platinum–carbon (Pt/C) catalyst, integrated into an MEA using a Nafion 117 membrane. This design is distinguished by a compact coin–cell configuration, incorporating precise signal conditioning and an electronic protection circuit. These features ensure rapid detection (response time < 1 s), high sensitivity, and enhanced safety, addressing critical industry challenges. The use of state-of-the-art materials, including highly porous carbon supports for catalyst dispersion, ensures optimal sensitivity and cost efficiency. Additionally, the integration of advanced signal conditioning circuits, including an INA111 operational amplifier, enhances the sensor’s precision. This approach enables the sensor to achieve high-performance metrics while remaining cost-effective.

The final chapter evaluates the sensor’s performance, particularly regarding its ability to operate efficiently at room temperature and response time, which are essential features for hydrogen safety monitorization in diverse environments. Testing across a hydrogen concentration range of 1–20% demonstrates the sensor’s rapid response and stable outputs. The integration of a signaling and protection module enhances the sensor’s reliability in real-world applications.

## 2. Materials and Methods

### 2.1. Materials and Apparatus

Toray Graphite Paper TGPH–090, 20% wet proofing, with a pore volume of 1.3 cm^3^/g, thickness of 0.28 mm, and 78% porosity, from FuelCellStore (Bryan, TX, USA). NAFION 117 membrane, which is based on a polytetrafluoroethylene (PTFE) backbone with side chains containing ether groups and a sulfonic acid unit at its end, with a thickness of 0.183 mm and >0.90 meq/g exchange capacity, from FuelCell Store. Catalyst type Vulcan XC72 40% Pt as a sensing material, with crystallite size of 2–3 nm and specific surface area of 70 m^2^/g, from FuelCellStore. ABG expanded graphite of high purity type ABG 1010, with a total specific surface of 30 m^2^/g, particle size content of less than 13 µm (95%), and Fe content < 100 ppm. Acetylene carbon black (NF) type Printex Kappa 100 PWD, with a total specific surface of 67 m^2^/g, particle size content of less than 45 µm (min 95%), and Fe content < 5 ppm. Activated carbon (AC) type WOS PL1000 M325, with a total specific surface of 1100 m^2^/g and a particle size content of less than 45 µm (min 80%). Spherical graphite (SG) type TOB-Graphite-T battery grade, high purity, with a total specific surface of 4 m^2^/g and particle size content of less than 23 µm min (95%). NAFION solution 5% (from Merck) in aliphatic alcohol and water (from DuPont) with ionic conductivity of ~1.23 × 10^−2^ Ω^−1^ cm^−1^. The H_2_ gas concentration required for the experiment was diluted to 5%, 10%, 15%, and 20% contents by mass flow controllers, with standard gas with argon (purity ≥ 99.999%).

### 2.2. Manufacturing of Sensing Element for Hydrogen Detection

The sensing element for hydrogen detection is made in the form of a coin and is based on the membrane electrode assembly (MEA) system, similar to a PEM fuel cell. The MEA system includes a cathode electrode, an ion-conducting membrane type Nafion 117, and an anode electrode, which were mounted by hot pressing in the form of a sandwich. Later, the MEA system was mounted in a cut CR2016 coin-type cell.

The preparation of the cathode element includes the following steps: 1 g of carbon material type ABG/NF/AC or SG was mixed manually with 1.5 mL of Nafion 5% solution for 2 min in a stainless steel container for pre-homogenization, which then was inserted into a vacuum oven for degassing, followed by ultrasonication for 15 min. After ultrasonication, the mixture obtained was introduced into a mixing device with a vibrating table in a vacuum for 6 h at 400 rpm, at room temperature, finally obtaining a carbonic mixture in the form of a slurry. A sheet of Toray carbon paper (GDL) with a thickness of 200 µm is cut to 210 × 300 mm. The GDL sheet is attached to the vacuum bed of a Dr. Blade coating device. By setting the height of the device lamella compared to the GDL surface, a homogeneous layer of 100 µm thick cathodic slurry is deposited by the Dr. Blade method. After depositing the cathode material, the samples are left to dry in the oven at 80 °C, in the first stage, for 2 h in order to remove the solvent from the solvent content. In the second stage, the GDL foil with slurry is left to dry at 110 °C for 12 h.

To make the anode in the form of a slurry, the following procedure was used: 0.5 g of Vulcan XC72 40% Pt catalytic material mixed manually with 1 mL of Nafion 5% solution for 2 min in a stainless steel container for pre-homogenization, which then is inserted into a vacuum oven for degassing, followed by mechanical mixing in a vacuum with a stirrer at 200 rpm for 1 h at room temperature, in a mixing device with a vibrating table, finally obtaining a catalytic mixture in the form of a slurry. A layer of 200 µm thick slurry is deposited on a Toray carbon paper by setting the height of the Dr. Blade blade, following the procedure applied for the preparation of the cathode element.

**Figure 1:**
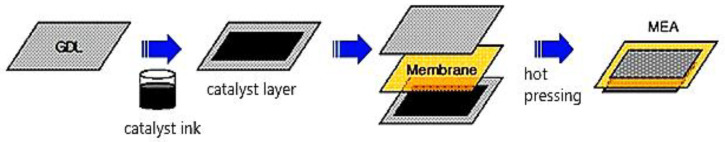
Illustration of the manufacturing process of the MEA assembly.

After drying, the carbon paper with the deposited slurry layer is positioned in a cutting device to cut the carbon-based positive-anode electrode to 19 mm in diameter and, respectively, the Pt/C-based negative-cathode electrode to 16 mm. The Nafion membrane is cut into discs with a diameter of 20 mm. Next, the positive electrode, the proton exchange membrane, and the negative electrode are sandwiched between two Teflon plates in a MEA assembly. The MEA assembly is further hot pressed with 1 tf at a temperature of 140 °C, after which the assembly is allowed to cool down slowly, maintaining the pressure. The diagram of the MEA manufacturing process is presented in Figure 1.

**Figure 2:**
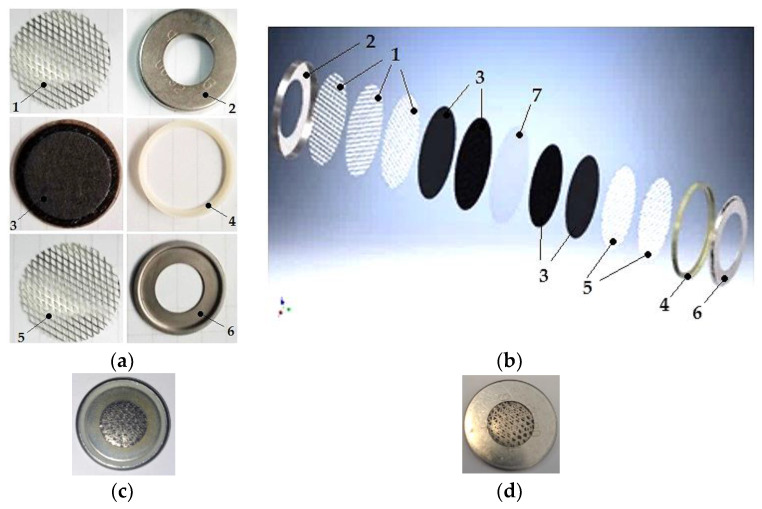
The MEA assembly mounted in a coin cell CR2016 housing, forming the sensitive element in the PEM configuration: (**a**) the component parts of the PEM sensitive element; (**b**) the layout of the components in the PEM sensitive element in an exploded view; (**c**) the PEM-sensitive element in the CR2016 coin cell case; (**d**) the PEM-sensitive element in the CR2016 coin cell case, in reverse side.

The MEA assembly is later mounted in a coin cell CR2016 housing, thus forming the sensitive element in the PEM configuration. The component parts of the PEM-sensitive element are shown in Figure 2a. With the help of an ultrasonic welding device, nickel meshes are cut and welded to the corresponding covers of the cut CR2016 coin cell in order to obtain a sandwich thickness of nickel mesh/cathode/membrane/anode/nickel mesh as close as 1200 µm. Figure 2b represents the layout of the components in the PEM-sensitive element in an exploded view. The sandwiched components of the PEM-sensitive element are mounted into the CR2016 coin cell housing and manually pressed at 1500 psi (105 kgf/cm^2^) in an embossing mold, thus obtaining the PEM-sensitive element in the CR2016 coin cell case (Figure 2c,d). After embossing, the sensitive element is subjected to the testing process.

The component parts of the PEM sensitive element (Figure 2a,b) arranged in the CR2016 coin cell case are the following: 1—nickel mesh for the positive electrode; 2—large steel casing type CR2016; 3—GDL/Vulcan XC72 40%, Pt/C−electrode assembly, and GDL/C−electrode assembly; 4—separator gasket; 5—nickel mesh for the negative electrode; 6—small steel casing type CR2016; 7—Nafion membrane.

To summarize, the commercial Vulcan XC72 40% Pt nanoparticles are combined in situ with a carbon porous substrate and solid polymer electrolyte to create a solid-state polymer electrochemical hydrogen sensor. To ensure excellent contact between the materials, the hot-pressing method is employed. Through this method, the Nafion membrane is attached to the sensing catalytic materials like C (cathode) and Pt/C (anode). As a result, Pt/C/Nafion/C-based sensing elements are fabricated and assembled into an integrated H_2_ sensor within a modified CR2016 coin cell. Subsequently, the sensing properties of the Nafion-based amperometric hydrogen sensors are investigated under varying H_2_/Ar mixtures at concentrations of 5%, 10%, 15%, 20%, and 100% hydrogen. This assessment helps in understanding how the sensor responds to different hydrogen concentrations and validates its performance in various scenarios.

## 3. Discussion

### 3.1. Overview Regarding the Manufacture of Amperometric Sensors Based on Nafion Membranes

The present study introduces an innovative amperometric electrochemical hydrogen sensor designed to detect hydrogen leaks efficiently and accurately at room temperature. The sensor utilizes a Nafion membrane integrated with platinum–carbon catalysts, forming a solid-state structure with high sensitivity and selectivity for hydrogen detection.

The carbon-based electrodes have been verified to possess a uniform current density [32] and are well-suited for electrochemical gas sensors due to their affordability and ease of fabrication [33]. Hence, it is essential to develop a simple and cost-efficient method for modifying carbon electrodes. The platinum–carbon catalyst produced by dispersing platinum nanoparticles on activated carbon offers several advantages, including high reactivity, even distribution, reduced usage of precious metals, and cost-effectiveness. Consequently, this catalyst maintains a heightened sensitivity in sensors while lowering fabrication expenses, which is crucial for advancing the sensor’s commercial development.

Pt-Carbon has found extensive use as an electrocatalyst in various applications, including fuel cells [34,35], hydrodeoxygenation [36], and hydrogen production from water [37,38]. Nevertheless, its main limitation lies in its high cost. Therefore, substantial efforts have been devoted to developing Pt-free electrocatalysts for many years [39,40,41]. For example, researchers like V. Singh Bhati et al. [42] and J.-H. Kim et al. [43] have successfully modified ZnO nanostructures and nanowires, respectively, to enhance their response to hydrogen in higher temperature resistive heated MOX sensors. Pd modifications have demonstrated improved discrimination toward NO_2_ and organic compound vapors [43], while nanoporous fibers loaded onto ZnO have shown similar improvements in hydrogen response [42]. Although these materials perform exceptionally well at elevated temperatures (>150 °C), Lupan et al. have achieved a room-temperature, single-bridge Au nanoparticle-decorated ZnO nanowire with high sensitivity to hydrogen and well-defined sensitivity to humidity [44].

Utilizing polymer solid electrolytes in the mentioned type of sensors enables lower temperature operation due to their high proton conductivity, even at reduced temperatures [45,46,47]. However, the materials commonly used in these devices are mostly limited to Nafion [48]. When fabricating amperometric sensors based on Nafion membranes, achieving excellent contact between the Nafion membrane and catalytic materials is crucial to ensure good proton and electronic conductivity [3]. The recent literature has reported various sensors based on Nafion [49], most of which are designed for detecting hydrogen [50,51], carbon monoxide [52,53], and oxygen [54,55].

In the international literature, various performance criteria are specified for different types of hydrogen sensors. These criteria include reliable response, fast response and recovery time (less than 1 s), low cross-sensitivity, low power consumption (less than 100 mW), and small size [56,57,58]. The most critical indicators of hydrogen sensors are sensitivity, response time, recovery time, and selectivity. Sensitivity refers to the slope of the relationship curve between the sensor signal value and the concentration of hydrogen. It characterizes how well the sensor can detect varying hydrogen concentrations. Response time is defined as the duration it takes for the sensor signal to reach 90% of its stable response value when exposed to hydrogen, while recovery time is the time for the sensor signal to return to 10% of its stable response value after the hydrogen source is removed. Selectivity is another essential factor, which is expressed by the ratio of the sensor’s response value or sensitivity to hydrogen compared to other interfering gases. A highly selective sensor will respond primarily to hydrogen and minimize responses to other gases, ensuring accurate and reliable hydrogen detection. The electrode material used in hydrogen sensors must meet specific requirements, including (i) high catalytic activity for the target gas’s electrode reaction, enabling efficient sensing of hydrogen; (ii) high electrical conductivity, facilitating rapid electron transfer during the sensing process; (iii) stability at the operating temperature of the sensor to ensure consistent and reliable performance; (iv) suitable porosity and gas hole size to allow for unhindered diffusion of hydrogen molecules to the electrode surface, optimizing sensitivity and response times [56].

### 3.2. The H_2_ Sensing Mechanism

The sensor is a fuel cell-based amperometric sensor. The H_2_/Ar mixture is passed to the sensing part (anode), and the oxygen is passed to the counter electrode (cathode). The hydrogen oxidation reaction is expressed by Equation (1) (Jaianthia et al., 2018 [59]):

(1) $$H_{2}\to 2H^{+}+2e^{-};E^{0}=0.00\;V$$

The hydrogen oxidation reaction occurs at the surface of the carbon-supported Pt nanocatalyst, where the hydrogen spillover phenomenon occurs. Hydrogen spillover is a surface-based process in which hydrogen atoms, produced by the decomposition of H_2_ on a metal nanoparticle, move to another location on the catalyst that has a site capable of accepting hydrogen (Shen et al., 2022 [60]). This phenomenon involves the interaction between the active hydrogen atoms and the carbon-based support material. It begins with the dissociation of H_2_ molecules at the metal sites. Subsequently, the activated hydrogen atoms travel to the adjacent support surface through the proton-conducting Nafion membrane, where protons (H^+^) diffuse toward O^2−^ anions, forming O–H and H–O–H bonds while also generating electrons (e^−^) that reduce nearby catalytic sites close to the O–H bonds (Shen et al., 2022; Yamazaki et al., 2021 [60,61]). On the metal free-carbon electrode (cathode) operated in the air, the oxygen reduction reaction (ORR) proceeds through a sluggish two-electron pathway in two steps to complete the reduction in O_2_ molecules to OH^−^ ions. In an acidic environment (e.g., Nafion electrolyte), the two-step 2e pathway includes the following steps (Equations (2) and (3), Ma et al., 2019 [62]):

(2) $$O_{2}+{2H}^{+}+{2e}^{-}\to {2H}_{2}O_{2};\;E^{0}=0.69\;V\;vs.\;SHE$$

(3) $$H_{2}O_{2}+{2H}^{+}+{2e}^{-}\to {2H}_{2}O;\;E^{0}=1.776\;V\;vs.\;SHE$$

Although the carbon surface tends to favor a two-electron oxygen-reduction reaction pathway due to its relatively weak binding with oxygen intermediates, the cathodic overpotential can drive the reaction to produce water (H_2_O) instead, significantly reducing the selectivity and production rate of hydrogen peroxide (H_2_O_2_), especially in acidic environments. This is because, under cathodic potentials in acids, the catalyst surface accumulates a high concentration of protons, which are likely to further reduce the H_2_O_2_ molecules produced locally to H_2_O (Zhang et al., 2022 [63]). The general reaction is presented in Equation (4) (Ma et al., 2019 [62]):

(4) $$H_{2}+\frac{1}{2}O_{2}\to H_{2}O;\;E^{0}=1.229\;V$$

### 3.3. The Signal-Conditioning Electronic Block

The signal conditioning electronic block taken from the H_2_ sensitive element was made from the following electronic modules:

- The module of differential voltage sources, stabilized, isolated, and protected from surges or overcurrent;
- The electronic conditioning module of the signal taken from the H_2_ sensitive element;
- The electronic protection and signaling module.

#### 3.3.1. The Module of Differential Voltage Sources, Stabilized, Isolated, and Protected from Surges or Overcurrent

**Figure 3:**
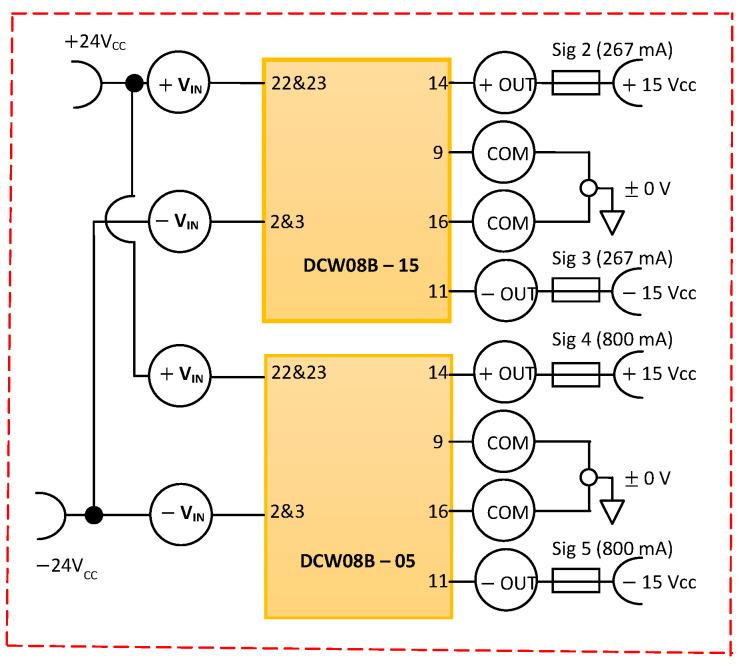
The electronic circuits of ±15 V and ±5 V differential voltage sources.

To power the signal-conditioning electronic block from the sensing element of H_2_ without having “ground loops”, it is necessary to use two differential DC voltage sources, stabilized, isolated, and protected. For this purpose, a differential integrated DC voltage source of ±15 V_DC_ was used, as well as a differential integrated DC voltage source of ±5 V_DC_ (Figure 3). By using these integrated power supplies (Figure 3), the signal conditioning electronic block from the sensing element of H_2_ is supplied with power from a single industrial DC voltage-supply voltage source of +24 V_DC_.

We consider the currents absorbed by each individual differential source to be maximum I = 200 mA, both for each plus source and each minus source. The most natural way to achieve these requirements is to use the DCW08B–15 and DCW08B–05, both from Mean Well Enterprises Co. LTD. (New Taipei City, Taiwan), isolated DC-DC converter (Figure 3). Some of the specifications of the DCW08B–15 isolation DC-DC converter are the convection cooling method, physical dimensions of 1.25″ × 0.80″ × 0.48″, 80% efficiency, 1000 V_DC_ isolation, two outputs, operating temperatures between −40, …, +71 °C, 0.267 A output current for both outputs, a nominal input voltage of 18, …, 36 V_DC_, a nominal output voltage of ±15 V_DC_, and a power of 8 W. Each individual differential source is additionally protected against short-circuit by means of 315 mA fuses (Figure 3).

#### 3.3.2. The Electronic Conditioning Module of the Signal Taken from the H_2_ Sensing Element

**Figure 4:**
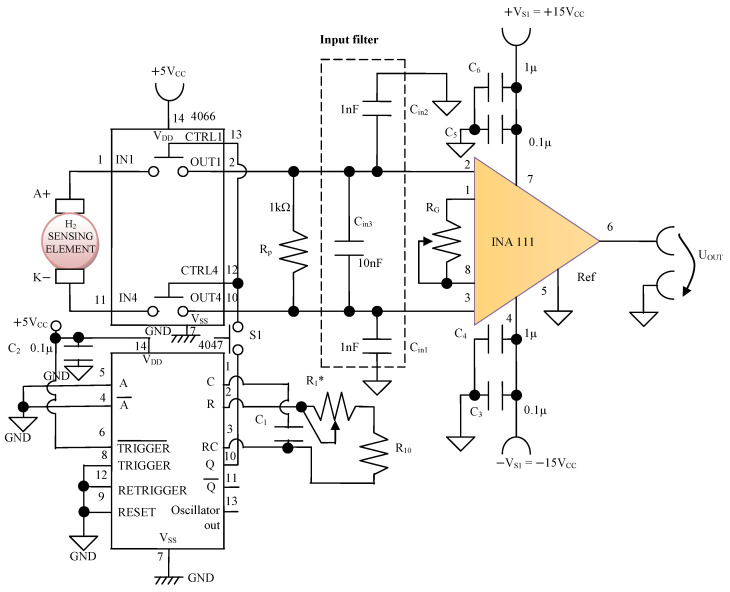
The electronic conditioning circuit of the signal taken from the H_2_ sensing element.

The electronic conditioning module of the signal taken from the H_2_ sensing element (Figure 4) consists of an instrumentation amplifier, INA111, with a high common mode rejection ratio (CMRR) of at least 106 dB and bias currents of at most 20 pA. This instrumentation amplifier is made with an input circuit with FET transistors in the “differential input mode” connection to be able to accurately pick up the signals from the sensitive element. By using an INA111 high-speed FET-Input instrumentation amplifier, a very good dynamic performance is ensured, like a slew rate of 17 V/µs. Setting the value of the amplification factor for the instrumentation amplifier INA111 to A = 5 is achieved by adjusting the external resistor R_G_ (Figure 4) at the value R_G_ = 12.4 kΩ. A low-pass filter placed at the input of INA111 is made of capacitors Cin1, Cin2, and Cin3. The values of these capacitors are found in the relationship C_in1_ = C_in2_ and C_in3_ = 10 C_in1_ to improve the CMRR. The input impedance of the INA111 amplifier is extremely high, approximately 10^12^ Ω, having the consequence of minimizing the distortion of the received signal.

The digital electronic circuits CD 4066 and CD 4047, respectively, (Figure 4) perform the intermittent connection of the H_2_ sensing element to the electronic conditioning circuit with a time constant given by τ = 4.4 C_1_ (R_10_ + R_1_*) in order to facilitate the optimization of absorption/desorption processes. The CD 4047 integrated circuit is configured as an astable multivibrator. The time constant τ can be adjusted in the range of τ = 8 s, …, 30 s. In this way, equal times are ensured for the absorption/desorption processes in the range of 4 s, …, 15 s.

#### 3.3.3. The Electronic Protection and Signaling Circuit

**Figure 5:**
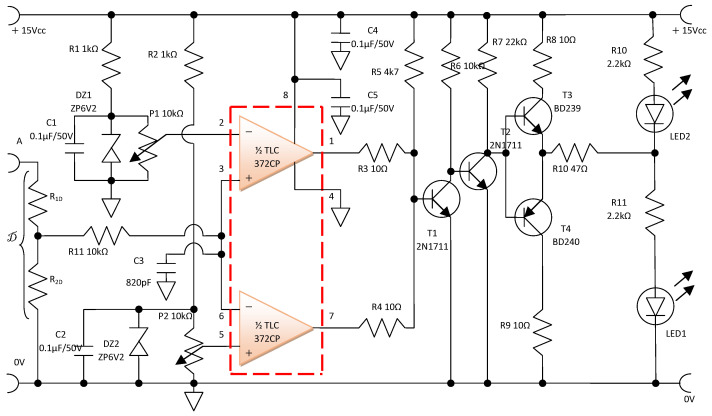
The electronic protection, signaling, and control circuit.

The electronic protection and signaling circuit (Figure 5) takes the common-mode voltage from the output of the electronic conditioning circuit (Figure 4) by means of the voltage divider D, made with resistors R_1D_ and R_2D_. The purpose of integrating the electronic protection and signaling circuit is to identify the exceeding of a predetermined dangerous H_2_ concentration and to signal this situation. More than that, the signal that ensures the signaling function can be used to activate a static relay with the command function. A static relay can be used to operate safely in an explosive environment and to command the shutdown of the H_2_ supply, as well as the activation of a ventilation system.

In order to realize the electronic protection and signaling circuit (Figure 5), a specialized integrated circuit, TLC 372 CP, is used in a window comparator connection. The operation of the electronic protection and signaling circuit (Figure 5) comprises two defined adjustable voltage thresholds, the lower threshold VDL and the upper threshold VDH. These thresholds are defined by the components R2, DZ2, P2, and C2 respectively to R1, DZ1, P1, and C1 (Figure 5).

Resistors R1 and R2 perform the operation of Zener diodes DZ1 and DZ2 in the third quadrant, limiting the current through them to 20 mA.

The electric potentials collected from the potentiometers P1 and P2 are applied to the inverting input of the ½ TLC 372 HP, respectively, to the non-inverting input of the ½ TLC 372 HP, which operates in a window comparator connection.

The potential from the output of the window comparator pins 1 and 7 is applied to the base of the bipolar transistor T1. The structures T1, T2, T3, and T4, together with the related components, apply a potential that can be used for protection control and/or signaling.

Two situations are distinguished in the operation of this electronic circuit:

- If the potential at the input of the window comparator is below VDL or above VDH, a potential equivalent to 0 Logic, in positive logic, will be provided on resistor R10 with respect to the reference potential;
- If the potential at the input of the window comparator is above VDL and below VDH, a potential equivalent to 1 Logic, in positive logic, will be provided across resistor R10 with respect to the reference potential.

The potential at the input of the window comparator is collected through a resistive divider, D (see Figure 5).

**Figure 6:**
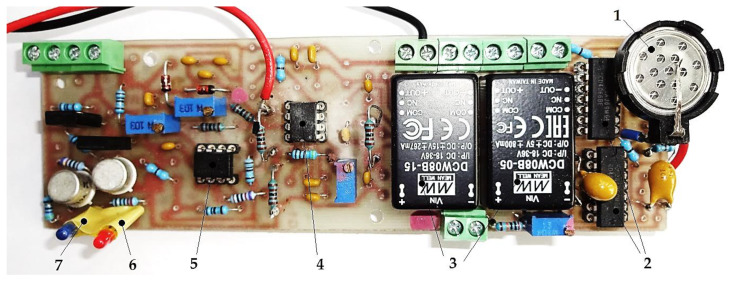
The H_2_ sensor, together with the electronic protection module; practical implementation.

The experimental–functional model of the amperometric electrochemical sensor for hydrogen detection was made by assembling the PEM coin-type sensitive element on the electronic board, which also includes the electronic block for conditioning the electrical signal taken from the sensitive element (Figure 6).

Figure 6 shows the practical implementation of the H_2_ sensor together with the electronic protection module. The main components of this module (Figure 6) are 1—the sensing element for hydrogen detection; 2—digital electronic circuits CD 4066 and CD 4047; 3—DCW08B–15 and DCW08B–05 isolated DC-DC converter; 4—instrumentation amplifier INA111; 5—integrated circuit, TLC 372 CP, that is used in a window comparator connection; 6—the red LED, enabled when potential at the input of the window comparator is above VDL and below VDH; 7—the blue LED, enabled when the potential at the input of the window comparator is below VDL or above VDH.

### 3.4. Laboratory Testing

The present study introduces an innovative amperometric electrochemical hydrogen sensor designed to detect hydrogen leaks efficiently and accurately at room temperature. The sensor utilizes a Nafion membrane integrated with platinum–carbon catalysts, forming a solid-state structure with high sensitivity and selectivity for hydrogen detection.

**Figure 7:**
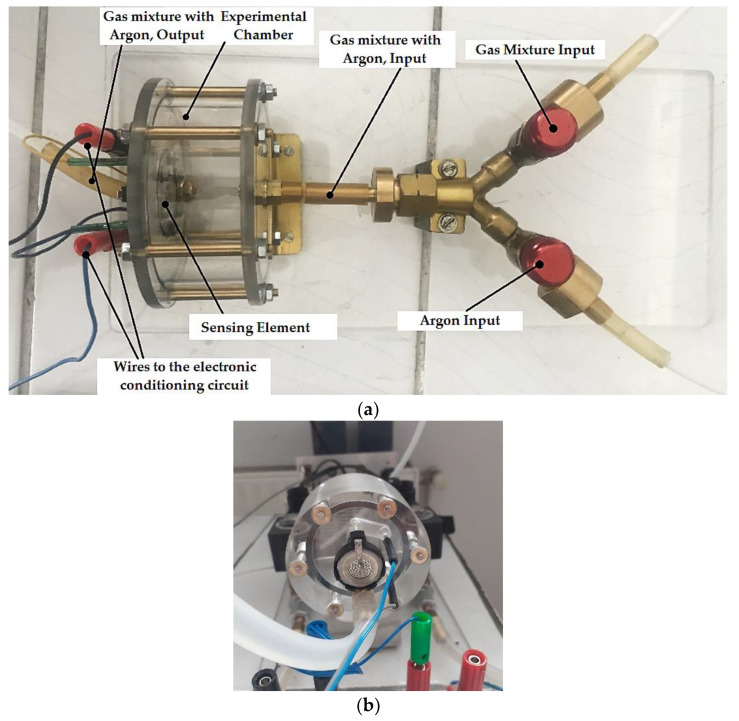
The device for laboratory testing of Nafion-based amperometric hydrogen sensors, practical implementation: (**a**) the components of device; (**b**) the positioning of the holder for the coin-type sensing element.

The sensing properties of the coin sensor were investigated in various H_2_/Ar mixtures at concentrations of 1%, 5%, 10%, 15%, and 20% hydrogen. This evaluation helps to understand how the sensors respond to different hydrogen concentrations and validates their performance in different scenarios. For laboratory testing of Nafion-based amperometric hydrogen sensors, a device shown in Figure 7a,b is used. This device consists of a sealed measurement chamber where the gas containing different concentrations of hydrogen mixed with argon is introduced, a holder for the coin-type sensing element (Figure 7b) positioned on the wall of the measurement chamber, and valves to control the flows of hydrogen mixed with argon, as well as to be able to purge pure argon after each measurement (Figure 7a). The electrical outputs were monitored and registered with a data logger.

The tests were performed under laboratory conditions, with a relative humidity of atmospheric air of 50% RH and a temperature of 20 degrees Celsius.

**Figure 8:**
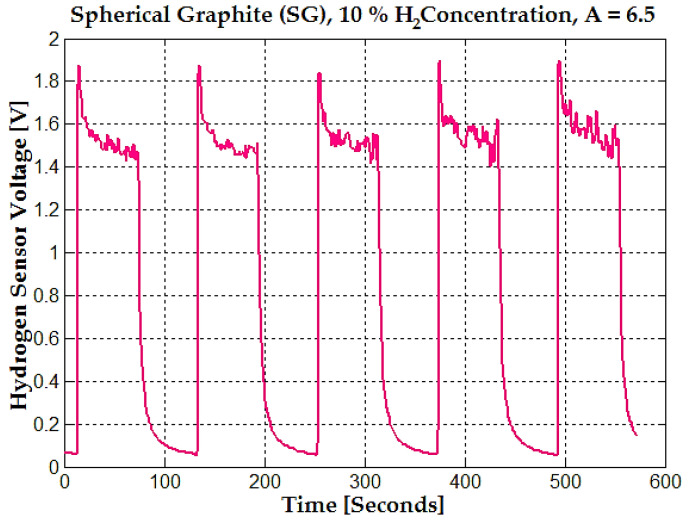
The time response of the hydrogen sensor voltage for spherical graphite (SG) at 10% hydrogen concentration (magenta line), with an amplification factor of A = 6.5.

The sensor voltage was subjected to a repetitive cycle over time for 10% hydrogen concentration (Figure 8).

This cycle represents the alternating exposure of the sensor to hydrogen gas and, respectively, the removal of hydrogen gas. At 10% hydrogen concentration, the sensor stabilizes at 1.5–1.6 V; the average values are considered under the voltage value of the rapid transient response for the spherical graphite material.

**Figure 9:**
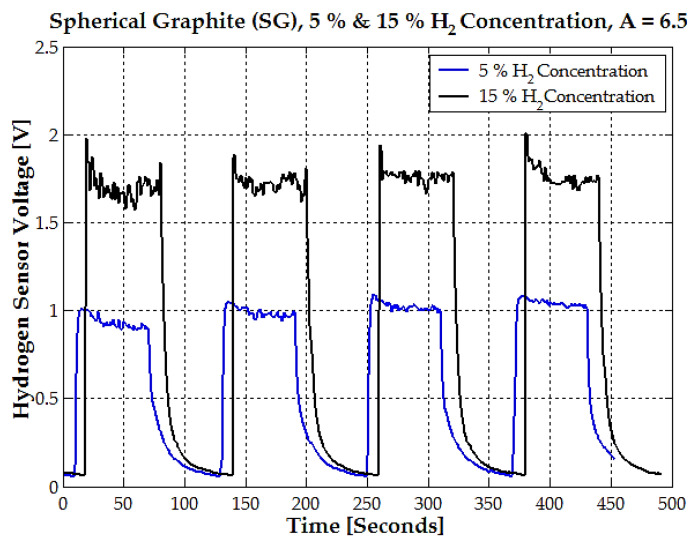
The time response of the hydrogen sensor voltage for spherical graphite (SG) at 5% and 15% hydrogen concentrations (blue and black lines), with an amplification factor of A = 6.5.

The sensor voltage was subjected to a repetitive cycle over time for both 5% and 15% hydrogen concentrations (Figure 9). This cycle represents the alternating exposure of the sensor to hydrogen gas and, respectively, the removal of hydrogen gas. At 5% hydrogen concentration, the sensor stabilizes at an average voltage of 1.05 V during the “on” phase. At 15% hydrogen concentration, the sensor stabilizes at a higher average voltage of 1.8 V during the “on” phase.

**Figure 10:**
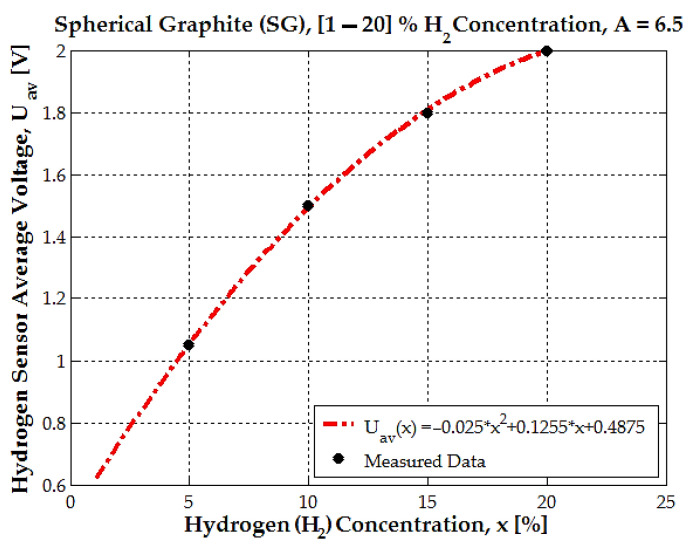
The dependence between the hydrogen sensor’s average voltage (*U_av_*) and hydrogen concentration *x* for spherical graphite (SG), with an amplification factor of A = 6.5.

This graph, from Figure 10, illustrates the relationship between the hydrogen sensor’s average voltage *U_av_* and hydrogen concentration *x* for spherical graphite (SG), with an amplification factor A = 6.5. The black dots represent the average measured data points for different hydrogen concentrations.

The dashed red line represents the quadratic fit to the experimental data and is represented by the following equation:

(5) $$U_{av}\left(x\right)=-0.025\cdot x^{2}+0.125\cdot x+0.487$$

The quadratic model (red dashed line, Figure 10) introduces a nonlinear behavior; the deviation from linearity increases as hydrogen concentration increases from 15% to 20%; the quadratic fit error is very small, 0.32%. The average voltage increases almost linearly from 1% to 10% hydrogen concentrations, from 0.6 V to 1.5 V. The nonlinear behavior is more accentuated between 15% and 20% hydrogen concentration, where the sensor output is 1.8 V for 15% H_2_ concentration and 2.04 V for 20% H_2_ concentration. With hydrogen concentration above 20%, the sensor output response decreases under 2 V for an amplification factor of A = 6.5.

**Figure 11:**
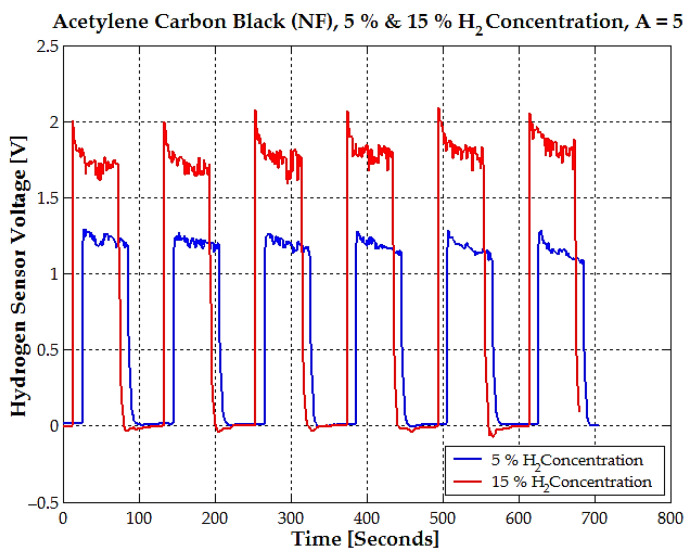
The time response of the hydrogen sensor voltage for acetylene carbon black (NF) at two hydrogen concentrations, 5% (blue line) and 15% (red line), with an amplification factor A = 5.

The sensor voltage exhibits a repetitive cycle over time for both 5% and 15% hydrogen concentrations (Figure 11).

This cycle corresponds to alternating exposure of the sensor to hydrogen gas (“on” phase) and the removal of hydrogen (“off” phase), allowing it to reset. At 5% hydrogen concentration, the sensor stabilizes at an average voltage of approximately 1 V during the “on” phase. At 15% hydrogen concentration, the sensor stabilizes at a higher average voltage of about 1.8 V during the “on” phase.

**Figure 12:**
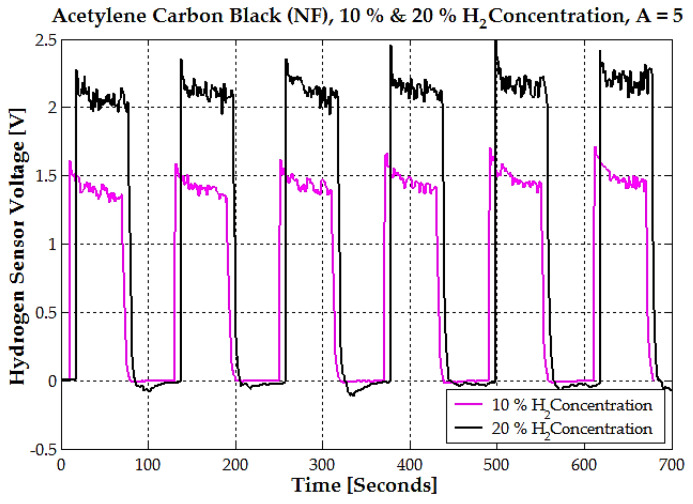
The time response of the hydrogen sensor voltage for acetylene carbon black (NF) at two hydrogen concentrations, 10% (magenta line) and 20% (black line), with an amplification factor of A = 5.

**Figure 13:**
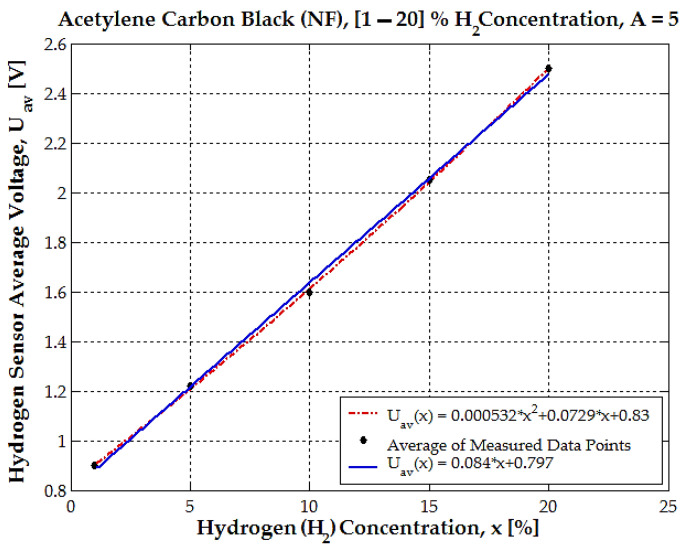
The dependence between the hydrogen sensor’s average voltage (*U_av_*) and hydrogen concentration (*x*) for acetylene carbon black (NF), with an amplification factor of A = 5.

In the “off” phase, hydrogen is removed, and the voltage for both concentrations drops to nearly 0 V, indicating effective sensor recovery. The voltage levels observed here for 5% (approx. 1.2 V) and for 15% (approx. 1.8 V) align closely with the linear/quadratic fits from the concentration vs. voltage graph (Figure 11, Figure 12 and Figure 13). This consistency indicates that the sensor’s voltage output is both predictable and reliable for these concentrations. The consistent voltage levels and repeatable responses suggest minimal errors for 5% and 15% concentrations.

The sensor voltage has a repetitive cycle over time for both 10% and 20% hydrogen concentrations (Figure 12). As seen previously, this cycle corresponds to alternating exposure of the sensor to hydrogen gas and the removal of hydrogen. At 10% hydrogen concentration, the sensor stabilizes at an average voltage of approximately 1.6 V during the “on” phase. At 20% hydrogen concentration, the sensor stabilizes at a higher average voltage between 2.2 V and 2.6 V during the “on” phases. This 0.4 V variation in the voltage peaks shows poor sensor stability at 20% hydrogen concentration.

This graph shown in Figure 13 illustrates the relationship between the hydrogen sensor’s average voltage *U_av_* and hydrogen concentration *x* for acetylene carbon black (NF) with an amplification factor A = 5. The black dots represent the average measured data points, showing the experimental sensor output for various hydrogen concentrations.

The dashed red line represents the quadratic fit to the experimental data calculated using the following equation:

(6) $$U_{av}(x)=0.000532\cdot x^{2}+0.0729\cdot x+0.83.$$

The blue line represents a linear fit with the following equation:

(7) $$U_{av}(x)=0.084\cdot x+0.797,$$

where the hydrogen concentration is measured in percent (%), and $U_{av}(x)$ is the hydrogen sensor’s average voltage measured in volts.

We consider the curve showing the dependence of sensor voltage as a function of hydrogen concentration (Figure 13). This curve is quasi-linear, and it is possible to calculate the sensitivity of the sensor. In this regard, the value for sensitivity will be obtained as $S=0.0845V/H_{2}\%$:

(8) $$S=\frac{∆U_{av}}{∆x}=\frac{2.512-0.905}{20-1}≅0.08\;V/H_{2}\%$$

Both the linear and quadratic fits align closely with the measured data points across the range of hydrogen concentrations *x* from 1% to 20%. The quadratic model (red dashed line) introduces a slight curvature to account for any nonlinear behavior, but the deviation from linearity is minimal; as seen in the nearly identical trajectories of the two fits, the quadratic fit error is 0.51% only. The average voltage increases linearly (or nearly linearly) with hydrogen concentration, ranging from approximately 0.8 V at <1% hydrogen to around 2.5 V at 20%. Both the linear and quadratic models provide good approximations of the experimental data.

The quadratic fit accounts for slight deviations, but the linear fit is nearly sufficient for practical purposes, introducing an error of a maximum 2–3%. For applications that require very high accuracy, the quadratic model should be preferred. For simpler implementations with tolerable error margins, the linear model might suffice, especially for hydrogen concentrations below 15%.

**Figure 14:**
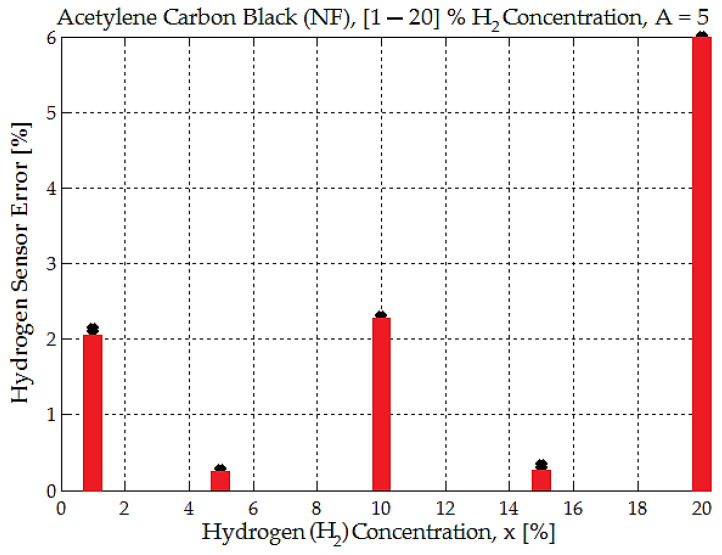
The error arises from the mismatch between measured data and reference values for the linear variation model for the acetylene carbon black (NF) material.

Several conclusions can be extracted from the above error graph (Figure 14). The error is not constant and varies significantly across the range of hydrogen concentrations. Lower hydrogen concentrations (1–15%) have relatively lower errors compared to higher concentrations (e.g., 20%). For the specific data points, at 1% hydrogen concentration, the error is approximately 2% and decreases sharply to nearly 0.3% at around 5% concentration. At 10% concentration, the error increases again to about 3%. At 15%, the error decreases to a very low value (<1%). At 20% concentration, the error spikes drastically to its maximum value of around 6% (or even 20% for some measurements).

The previous error arises from the mismatch between the measured data and the expected values based on a grade-two (quadratic) equation and a linear variation model. The increasing error at higher hydrogen concentrations implies that neither the quadratic model nor the linear model accurately captures the behavior of the sensor at these levels.

There is a difference of 10 to 20 mV between all measured data points of the sensing element and the average reference value. With the electronic conditioning scheme, this error can be amplified by the amplification factor A = 5 or A = 6.5, resulting in a voltage difference of 0.05 to 0.1 V. A measurement error of 3% to 6% was calculated for each hydrogen concentration. However, for a 20% hydrogen concentration, the measurement uncertainty is significantly larger, with errors ranging from 6% to 20% observed in the experimental data compared to the reference value of 2.5 V.

The sensor demonstrates good accuracy for low to moderate hydrogen concentrations (1–15%) but struggles to maintain accuracy at higher concentrations (20%). The quadratic and linear models perform acceptably for intermediate concentrations, between 1% and 15%.

Table 1 shows the electrical outputs in voltage generated by the coin sensor at different concentrations of hydrogen (1% and 5% to 20%) while fed with a mixture of H_2_/Ar on the anode side and environmental air on the cathode side.

**Figure 15:**
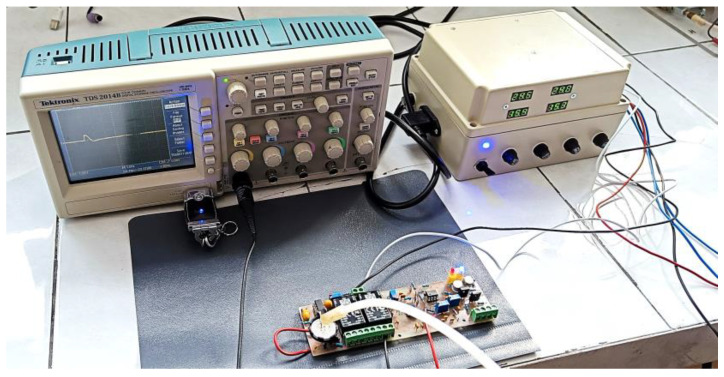
Testing of the sensor for hydrogen detection, together with the electronic protection module.

Subsequently, to view the response time of the hydrogen detection sensor, a Tektronix TDS 2014B digital oscilloscope from Tektronix (Beaverton, OR, USA) was used in recorder mode (Figure 15). The voltage variation over time from the output of the H_2_ sensor could be recorded on the oscilloscope screen and saved as a file with the extension “BMP” on a memory stick from the “USB” port of the oscilloscope.

**Figure 16:**
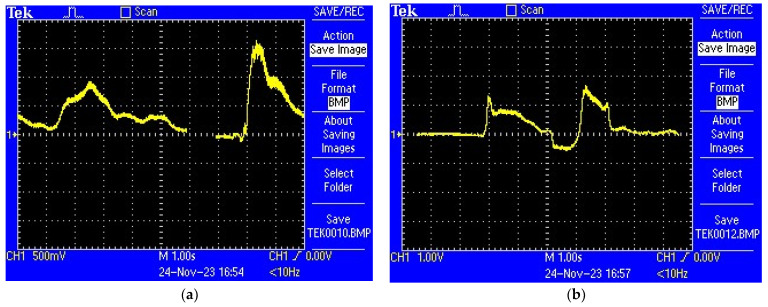
Testing of the sensor for hydrogen detection, two captures made with the Tektronix TDS 2014B digital oscilloscope, set in recorder mode: (**a**) sensor response to a 10% H_2_ concentration in air; (**b**) sensor response to a 15% H_2_ concentration, in air.

Hydrogen leakage in the laboratory environment was simulated. Hydrogen from four H_2_ cylinders, corresponding to concentrations of 5%, 10%, 15%, and 20%, was injected through a hose into the sensitive element area (Figure 15) and was detected by the H_2_ sensor. The captures made with the Tektronix TDS 2014B digital oscilloscope set in recorder mode are shown in Figure 16a,b.

Laboratory experiments confirmed the sensor’s quick response, with a maximum delay in the order of seconds, regardless of the tested hydrogen concentration (Figure 16). This rapid responsiveness is critical for real-time applications in safety-critical scenarios.

## 4. Conclusions

Selective gas sensors have proven effective for this purpose, like sensors with semiconductors, catalytic combustion, thermoelectric, chemiresistive, and electrochemical sensors [5,6,7,8,9,10,11,12,13]. In general, the progress of technology calls for cost-effective gas sensing devices that are both portable and quick to respond, Mostafa S. [10]. The last notable advancements in gas sensing have introduced an innovative detection method using field-effect transistors (FETs). For FET-based sensors, performance is assessed by the change in drain current as a result of a chemiresistive phenomenon. The interaction between the active material between the electrodes and gas molecules influences the electrical conductance, even at low gas concentrations, thereby directly converting it to an electrical signal [10]. However, FET-based sensors require a relatively biassed high voltage to the electrode’s terminals in order to evaluate the electrical current variations effectively.

Another proposed solution for cost-effective gas sensors, the electrochemical (potentiometric and amperometric) sensors, employing solid electrolytes, show great promise due to their compact design, straightforward detection mechanism, and high selectivity [3].

The electrochemical hydrogen sensor is operated in an amperometric mode, where a current is generated directly proportional to the concentration of the target analyte, hydrogen. One notable advantage of the amperometric gas sensor is that it does not require an internal heater, making it more suitable for use in potentially flammable environments compared to other types of sensors. Additionally, this sensor is known for its low power consumption, excellent selectivity, and linear response across a wide measurement range [64,65,66,67]. It operates by functioning similarly to a hydrogen fuel cell, where hydrogen and oxygen spontaneously react to produce an electric current in the sense of Faraday. The electrical current can be passed through a precision resistor, resulting in an easily measurable voltage directly proportional to the amount of hydrogen reacted.

An important characteristic of the H_2_ sensor is its ability to operate efficiently at room temperature without requiring a heating element. This characteristic enhances its safety in potentially flammable environments and contributes to its low power consumption.

Another noteworthy feature of the H_2_ sensor is its rapid response time, measured in the order of seconds, as demonstrated in all scenarios presented in laboratory experiments. This rapid responsiveness is critical for real-time applications in safety-critical scenarios. By employing composite catalyst material like Pt/C, the sensor achieves high reactivity and reduced use of precious metals. This approach minimizes manufacturing costs while maintaining excellent sensitivity, making it viable for widespread commercial applications.

The sensor demonstrates good accuracy for low-to-moderate hydrogen concentrations (1–15%) but struggles to maintain accuracy at higher concentrations (20%). The error is not constant and varies significantly across the range of hydrogen concentrations. Lower hydrogen concentrations (1–15%) have relatively lower errors compared to higher concentrations (e.g., 20%). At 1% hydrogen concentration, the error is approximately 2%; the error decreases sharply to nearly 0.3% at around 5% concentration. At 10% concentration, the error increases again to about 3%. At 15%, the error decreases to a very low value (<1%). At 20% concentration, the error spikes drastically to its maximum value of around 6%.

The quadratic and linear models perform acceptably for intermediate concentrations, between 1% and 15%.

The signal processing module, equipped with an INA111 instrumentation amplifier, ensures accurate and stable signal capture from the sensitive element. This electronic module includes a protection and threshold signaling circuit to enhance reliability and provide alerts for critical hydrogen levels.

In summary, the developed hydrogen sensor can offer a compact, safe, and efficient solution for hydrogen leakage detection, meeting critical requirements for sensitivity, selectivity, and response time. Future work may explore expanding the detection range and further reducing costs through alternative catalyst materials. These advancements could strengthen the sensor’s utility in the emerging hydrogen-based energy infrastructure.

## Figures and Tables

**Table 1:** Voltage [V] vs. Concentration [%] of H_2_ at A = 5.

Voltage [V] vs. Concentration [%] of H_2_ (A = 5)	1 [%]	5 [%]	10 [%]	15 [%]	20 [%]
ABG1010 Expanded Graphite	-	1.78	2.12	2.23	2.31
Activated Carbon (AC)	-	1.37	1.04	1.68	2.17
Spherical Graphite (SG)	-	0.80	1.15	1.38	1.57
Acetylene Carbon Black (NF)	0.905	1.22	1.60	2.05	2.51

## Data Availability

Data are contained within the article.
